# Supplementary material for: Modeling glioblastoma heterogeneity as a dynamic network of cell states
Source: Mol Syst Biol. 2021 Sep 16;17(9):e10105. doi: 10.15252/msb.202010105 (PMC8444284; doi:10.15252/msb.202010105)
Supplement: Supplementary file 6 — Source Data for Figure 5 [file MSB-17-e10105-s004.zip › Figure5A_sourcedata/GSEA_3071/nosigdb_stateII.GseaPreranked.1622795151171/DOROSZKO_U3054MG_CLUSTER_0.html]

Details for gene set DOROSZKO\_U3054MG\_CLUSTER\_0[GSEA]

|  || Dataset | state23071 |
| Phenotype | NoPhenotypeAvailable |
| Upregulated in class | na\_neg |
| GeneSet | DOROSZKO\_U3054MG\_CLUSTER\_0 |
| Enrichment Score (ES) | -0.6472689 |
| Normalized Enrichment Score (NES) | -2.9458957 |
| Nominal p-value | 0.0 |
| FDR q-value | 0.0 |
| FWER p-Value | 0.0 |
Table: GSEA Results Summary

  

Fig 1: Enrichment plot: DOROSZKO\_U3054MG\_CLUSTER\_0      
 Profile of the Running ES Score & Positions of GeneSet Members on the Rank Ordered List

  

| PROBE | GENE SYMBOL | GENE\_TITLE | RANK IN GENE LIST | RANK METRIC SCORE | RUNNING ES | CORE ENRICHMENT || 1 | MGLL |  |  | 227 | 0.848 | -0.0330 | No |
| 2 | ALCAM |  |  | 288 | 0.771 | -0.0269 | No |
| 3 | CTHRC1 |  |  | 449 | 0.651 | -0.0487 | No |
| 4 | ETS2 |  |  | 923 | 0.466 | -0.1527 | No |
| 5 | P2RX7 |  |  | 1383 | 0.360 | -0.2561 | No |
| 6 | L1CAM |  |  | 1402 | 0.357 | -0.2509 | No |
| 7 | PPP1R18 |  |  | 1691 | 0.312 | -0.3135 | No |
| 8 | COL15A1 |  |  | 1798 | 0.299 | -0.3315 | No |
| 9 | ARHGEF16 |  |  | 2046 | 0.267 | -0.3852 | No |
| 10 | COBL |  |  | 2311 | -0.261 | -0.4432 | No |
| 11 | SEMA6D |  |  | 2407 | -0.272 | -0.4592 | No |
| 12 | GPC4 |  |  | 2577 | -0.298 | -0.4929 | No |
| 13 | TMEM132B |  |  | 2623 | -0.304 | -0.4957 | No |
| 14 | PCBP4 |  |  | 2633 | -0.306 | -0.4896 | No |
| 15 | TSC22D4 |  |  | 2847 | -0.344 | -0.5329 | No |
| 16 | ADAM23 |  |  | 3033 | -0.380 | -0.5682 | No |
| 17 | CHRDL1 |  |  | 3219 | -0.432 | -0.6021 | No |
| 18 | PPP1R9A |  |  | 3366 | -0.486 | -0.6249 | No |
| 19 | PMEPA1 |  |  | 3390 | -0.494 | -0.6172 | No |
| 20 | FABP5 |  |  | 3513 | -0.549 | -0.6324 | Yes |
| 21 | OLIG1 |  |  | 3551 | -0.571 | -0.6260 | Yes |
| 22 | ERBB3 |  |  | 3561 | -0.578 | -0.6126 | Yes |
| 23 | CD9 |  |  | 3567 | -0.581 | -0.5981 | Yes |
| 24 | SHC3 |  |  | 3648 | -0.645 | -0.6004 | Yes |
| 25 | TRIM2 |  |  | 3688 | -0.672 | -0.5918 | Yes |
| 26 | PRUNE2 |  |  | 3709 | -0.693 | -0.5780 | Yes |
| 27 | PLCB1 |  |  | 3779 | -0.769 | -0.5741 | Yes |
| 28 | TTYH1 |  |  | 3807 | -0.795 | -0.5593 | Yes |
| 29 | AP002884.1 |  |  | 3858 | -0.849 | -0.5486 | Yes |
| 30 | SLAIN1 |  |  | 3862 | -0.855 | -0.5261 | Yes |
| 31 | BCHE |  |  | 3926 | -1.012 | -0.5143 | Yes |
| 32 | SOX2 |  |  | 3953 | -1.069 | -0.4917 | Yes |
| 33 | SOX6 |  |  | 3956 | -1.081 | -0.4629 | Yes |
| 34 | NCAM1 |  |  | 3966 | -1.106 | -0.4352 | Yes |
| 35 | PCSK1N |  |  | 3987 | -1.179 | -0.4082 | Yes |
| 36 | CDH2 |  |  | 3998 | -1.233 | -0.3773 | Yes |
| 37 | FAM181B |  |  | 4037 | -1.542 | -0.3449 | Yes |
| 38 | CADM1 |  |  | 4042 | -1.568 | -0.3034 | Yes |
| 39 | SORL1 |  |  | 4062 | -1.787 | -0.2597 | Yes |
| 40 | TUBB2B |  |  | 4063 | -1.810 | -0.2107 | Yes |
| 41 | COL9A3 |  |  | 4067 | -1.892 | -0.1602 | Yes |
| 42 | CKB |  |  | 4075 | -2.149 | -0.1037 | Yes |
| 43 | PTPRZ1 |  |  | 4097 | -4.027 | 0.0002 | Yes |
Table: GSEA details [plain text format]

  

Fig 2: DOROSZKO\_U3054MG\_CLUSTER\_0: Random ES distribution      
 Gene set null distribution of ES for **DOROSZKO\_U3054MG\_CLUSTER\_0**
